# Fusion of electromagnetic world and digital world through information metasurface: an interview with Tie Jun Cui

**DOI:** 10.1093/nsr/nwad199

**Published:** 2023-07-22

**Authors:** Linglong Dai

**Affiliations:** Linglong Dai (戴凌龙) is a professor at Tsinghua University

## Abstract

Metasurfaces consist of subwavelength artificial elements (also called meta-atoms) arranged in a two-dimensional fashion. Through careful design of these elements, the metasurfaces can control the amplitude, phase, frequency, and polarization of electromagnetic (EM) waves. Information metasurfaces extend this concept by characterizing the meta-atoms in a digital manner, integrating functionalities for information processing. The information metasurfaces can simultaneously manipulate the EM waves in reprogrammable ways and modulate the digital information in real time, making the fusion of the EM world and digital world. The information metasurfaces have found diverse applications in wireless communications, intelligent sensing and imaging, intelligent computing, and internet of things (IoT).

Prof. Tie Jun Cui is affiliated with Southeast University in Nanjing, China, where he serves as the Director of the State Key Laboratory of Millimeter Waves. He is a prominent researcher in the field of metamaterial, metasurface, and computational electromagnetics. He has proposed the concepts, and made systematic studies, of digital coding metasurfaces and programmable metasurfaces, and established the system of information metasurfaces, building a direct link between the electromagnetic world and digital world.

NSR spoke to Professor Cui on the recent advancement and the prospects of information metasurfaces.

## INFORMATION METASURFACES AND RECONFIGURABLE INTELLIGENT SURFACES

***NSR:*** Why did you propose digital coding metasurfaces, programmable metasurfaces, and information metasurfaces?

***Cui:*** In the past decades, the studies of metamaterials and metasurfaces have been conducted from the perspective of physics, and mainly focused on designing abnormal parameters of effective media (e.g. negative index of refraction), finding new physical phenomena (e.g. perfect imaging and generalized Snell's law), or realizing new functional devices (e.g. antennas and meta-lenses). I also followed the above model in my earlier researches on metamaterials. In 2014, I tried to rethink metamaterials from the perspective of information science (since I am from the area of information science and electrical engineering), and proposed to characterize meta-atoms by digital states ‘0’ and ‘1’ (with opposite phase responses) and control electromagnetic waves by encoding digital sequences on the space, resulting in the concept of digital coding of metamaterials and metasurfaces. All possible digital coding sequences and their functionalities can be pre-calculated and stored in a field programmable gate array (FPGA), then we realized the first programmable metamaterial and metasurface. Later, in 2018 and 2019, we further extended the space coding to time coding and space-time coding, and proposed time-domain-coding digital metamaterial/metasurface and space-time-coding digital metamaterial/metasurface to manipulate spatial beams, waveforms, and frequency spectra of electromagnetic waves both simultaneously and independently. The digital coding characterization makes metamaterials and metasurfaces evolve from

**Figure 1:**
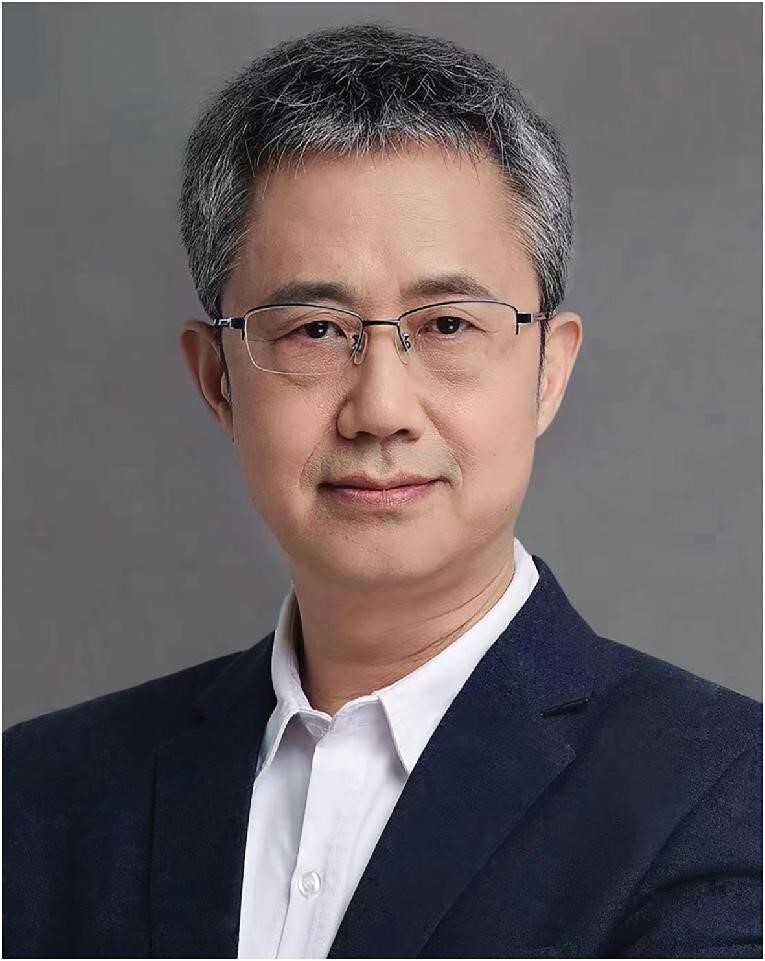
Professor Tie Jun Cui at Southeast University, China (*courtesy of Professor Tie Jun Cui*).

passive to active and from analog to digital, resulting in the capabilities to perform information operations and digital signal processing in the electromagnetic space. From this viewpoint, we proposed the concept of information metamaterials and metasurfaces, which can fuse the electromagnetic space and digital space together, and fulfill wave manipulations and information modulations simultaneously.

The concept of information metamaterials and metasurfaces can fuse the electromagnetic space and digital space together.

——Tie Jun Cui

***NSR:*** What are advantages of the information metasurfaces?

***Cui:*** The information metamaterials/metasurfaces have two advantages: (1) they can control waveforms, beam directions, and frequency spectra of electromagnetic waves in real time and in programmable ways; and (2) they can manipulate electromagnetic waves and modulate digital information simultaneously. The unique feature of information metamaterials and metasurfaces to fuse the electromagnetic space and digital space makes it possible to explore new theories (e.g. electromagnetic information theory) and build up new-architecture and/or simplified-architecture information systems for wireless communications, radar sensing, and microwave imaging.

***NSR:*** What is the relationship between information metasurface and reconfigurable intelligent surface?

***Cui:*** In 2019, several groups in the wireless communications society proposed a concept of reconfigurable intelligent surface (RIS) or intelligent reflecting surface (IRS), which can smartly reflect electromagnetic waves and beams to the desired directions and change the waveforms to enhance the coverage in real time. This concept is very attractive for wireless communications, since RIS can change the wireless environment from passive to active, engineer and enhance the wireless channels, and save electromagnetic energies. However, when RIS was proposed in the wireless communication community in 2019, it was only a virtual concept. Based on the first feature of the information metasurface, the programmable metasurface is a perfect platform to realize RIS since it can instantly control the electromagnetic beams, waveforms, and frequency spectra in a reprogrammable way. Due to this reason, we collaborated with Professor Shi Jin and made the first experimental verification of RIS to engineer the wireless environment by using the programmable metasurface. This is also a good example of collaboration between the societies of electromagnetic physics and wireless communication. In fact, the second feature of information metasurfaces will have more impact on wireless communications.

***NSR:*** What are the basic architecture and typical design methods of information metasurfaces and RIS? Which one is the most promising in your mind?

***Cui:*** The basic architecture of information metasurfaces typically consists of a planar arrangement of digital meta-atoms in subwavelength scales, which are designed to manipulate electromagnetic waves and modulate digital information. The meta-atoms can be metallic structures, dielectric resonators, and their combinations integrated with active devices (e.g. PIN diodes, varactor diodes, and MEMS), in which the active device will control their digital states. The design of information metasurfaces includes designing the digital meta-atoms (with phase coding, amplitude coding, or polarization coding) and metasurface array (or coding pattern) to achieve the desired functionalities. The typical design methods include analytical design, numerical simulations, optimization algorithms, and machine learning-based approaches. In my opinion, the machine learning-based approaches can be a promising direction in the future.

## WIRELESS APPLICATIONS AND BEYOND

***NSR:*** What are the important factors for information metasurfaces to attract extensive attention? What role can information metasurfaces play in the future? Can you give some examples?

***Cui:*** The information metasurfaces have gained extensive attention due to their capabilities to flexibly manipulate electromagnetic environments in a cost-efficient manner. The introduction of information metasurfaces into the communication systems has changed the paradigm from passively adapting the channel to actively controlling the channel. In the future, the information metasurfaces can greatly enhance the capacity of communication systems. For example, the information metasurfaces can provide extra reflecting paths of communications by beamforming, which can enhance the coverage of communication systems. In addition, by exploiting the ability of spatial modulations, the information metasurfaces can achieve more accurate localizations. The information metasurfaces have the potential to be used in many other fields thanks to their low cost, reconfigurability and programmability, and fusion of electromagnetic space and digital space.

***NSR:*** With the wide applications of information metasurfaces, what benefits can this technology bring to people's lives?

***Cui:*** The information metasurfaces can bring benefits in many aspects. With the capability to manipulate electromagnetic fields, the information metasurfaces can improve the quality of communication, especially in the places like basements, where the signals are usually highly attenuated. In addition, the information metasurfaces can realize spatial modulations and polarization modulations, which may change the paradigm of communications. Furthermore, the information metasurfaces or RISs, once widely deployed, are able to provide extra reflecting paths, which can enhance the capabilities of communication systems in sensing, detecting, and imaging.

***NSR:*** When do you think the information metasurface will become a WiFi-like reality for commercial use? What are the main challenges at present?

***Cui:*** I think that the information metasurface will become a WiFi-like reality in ∼15–20 years, however, there still exists some challenges. For example, the deployment of information metasurfaces requires synchronization with the current communication systems, which is not intuitive due to the passive characteristic of the information metasurfaces. The interference with signals at other frequencies also puts a challenge to the cooperation of different metasurfaces in the communication systems. What's more, the deployment of metasurfaces may introduce insecurity in the physical layer, which calls for rigorous protocol design. Despite the above challenges, the information

metasurface is still a promising technology and is very likely to become a WiFi-like reality in the future.

***NSR:*** Your research group has made enormous efforts in prototyping of information metasurfaces, for example, a metasurface-based transmitter. How does it differ from traditional transmitters in hardware and software design?

***Cui:*** Different from the conventional transmitters with high-cost and power-hungry RF components for baseband modulations, our developed metasurface-based transmitters do not need such components. By deploying the metasurfaces at the transmitter, the reflection-type phase-shifting can be exploited to replace the function of baseband modulation. Compared with the existing transmitter architectures, the information metasurface structure has much lower costs and lower power consumption. Besides, due to the fact that complex RF components are no longer needed, hardware complexity can also be reduced and the software design can be more flexible.

***NSR:*** For intelligent metasurfaces, how to understand ‘intelligent’? Does it mean that metasurfaces will be powered by artificial intelligence (AI)? What advances have been achieved in intelligent designs?

***Cui:*** From my perspective, intelligent metasurfaces have three-fold meanings. First, the reprogrammable capabilities of information metasurfaces can be integrated with the AI algorithms to accomplish intelligent tasks. For example, the combinations of information metasurfaces with convolutional neural networks (CNNs) have resulted in intelligent imaging, microwave cameras, and metasurface robotics. Second, the information metasurfaces can be smartly controlled by AI algorithms. These AI algorithms are capable of integrating multimodal information, including the wireless channel state information surrounding the metasurface, the visual information provided by deployed cameras, and the position or movement information of nearby objects gathered by various embedded sensors. With these kinds of information, the AI algorithm is capable of dynamically computing the optimal metasurface configuration to establish high-speed communication links, or to fulfill real-time sensing tasks. Third, the information metasurfaces can be used to build up programmable AI machines. For example, reprogrammable diffractive neural networks have been realized by using multi-layer information metasurfaces to fulfill the AI inference tasks, which can compute at the speed of light. These metasurface-based neural networks are expected to constitute future high-speed and low-power AI processors.

***NSR:*** As metasurfaces can change and control the information exchange, do they bring some security risks for personal information? If yes, how can we avoid these risks?

***Cui:*** Metasurfaces do bring challenges to information security, since they may be hijacked to direct the secret information towards eavesdroppers. However, this is very unlikely to happen because we can encrypt the metasurface controlling protocols. Rather than the risk of being hijacked, in fact, the metasurfaces are widely believed to be capable of enhancing transmission security. For example, by tuning the reflection coefficients of the metasurfaces rapidly, scrambling codes can be attached to the reflected signals to provide extra security to the reflected information. In addition, we can also minimize the received power of the eavesdropper by properly controlling the gain of a metasurface. By weaving the metasurfaces into electromagnetically invisible cloaks, personal information security can be further guaranteed by physically isolating the secrets from the environment.

## ONGOING EFFORTS AND FUTURE DIRECTIONS

***NSR:*** Metamaterial research has achieved great advances in the past two decades. As a leading expert in this field, could you forecast the road ahead for information metasurfaces? What breakthroughs will information metamaterials have in the future?

***Cui:*** Now we have witnessed the rapid progress of information metasurfaces in a number of applications like wireless communication, radar detection, and intelligent computation, where the corresponding system architectures have been greatly simplified, in the future, we can expect that more important roles can be played by the information metasurfaces in the above systems, since the direct signal processing capabilities during the wave-matter interactions will be greatly enhanced thanks to the advances of hardware. This will lead to deep integration of the digital and analog technologies, and open up a new perspective to manipulate waves and modulate information with low cost, simplicity and low power consumption. The integration of various information systems will also become much easier.

***NSR:*** Information metasurfaces have attracted increasing research interests both in wireless communications and electromagnetic fields, which gives birth to an interdisciplinary subject of electromagnetic information theory (EIT). Can you explain what it is and how it arose?

***Cui:*** Electromagnetic information theory is a unified framework that integrates the traditional Shannon information theory and Maxwell's equations. For wireless communications, the electromagnetic information theory aims to improve the effectiveness, safety and reliability of the system, and study the limit and approximation method of the system performance under physical constraints. The first-generation to the fourth-generation mobile communication systems have used time-frequency resources under the framework of Shannon information theory, and have achieved great successes. However, the Shannon

Information metasurfaces will lead to deep integration of the digital and analog technologies, and open up a new perspective to manipulate waves and modulate information.

——Tie Jun Cui

information theory is highly generalized and does not take into account the underlying physical processes. From a physical point of view, the essence of communication is a process wherein a series of physical quantities with separable states are detected and recognized at the receiving end after being transmitted through a noisy channel. The fifth- and future sixth-generation mobile communication systems use space resources and high carrier frequency signals to improve the transmission rate, and the research and design of these systems need to fully consider the physical propagation characteristics of the electromagnetic waves, which gives rise to the demand for new information theory. The information metasurface directly regulates the electromagnetic wave under the control of digital code stream, and thus it is a natural bridge connecting the information world and the physical world. Moreover, it is also a good starting point and landing point for researches of electromagnetic information theory. We are studying the electromagnetic information theory based on the information metasurface and have achieved several exciting results so far. We hope to provide a theoretical basis and practical guidance for designing new wireless systems based on the information metasurfaces in the future.

***NSR:*** Can you provide some advice for young researchers in this area?

***Cui:*** I am honored to provide some advice for the young researchers. First, we should pay special attention to multidisciplinary research to make our work more impactful. Information metasurface is a good example of multidisciplinary research, which involves electromagnetics, optics, materials science, and information science. Young researchers are quick to learn new knowledge and skills, so they should not only focus on their familiar topics, but watch what happens out of their comfort zones. Second, I hope young researchers can think outside the box to make groundbreaking discoveries. Instead of following the works from top experts in the field, we should try to take the lead, or even create a new research direction, which will be followed by other researchers instead. We should explore new ideas, propose innovative designs, and consider unconventional applications. Third, young researchers should understand that hardware is as important as software, and the coordinated design of hardware and software is usually a very powerful tool for challenging problems. Information metasurface is such an example, which heavily relies on both hardware and software. We should not emphasize software exclusively whilst ignoring hardware. Finally, young researchers must understand that academic integrity is the bottom line for all researchers. You should not set a time bomb for your future when you are still young. You may spend many years to build your reputation, but you just need one academic misconduct to destroy it.

